# Photo-Detectors Integrated with Resonant Tunneling Diodes

**DOI:** 10.3390/s130709464

**Published:** 2013-07-22

**Authors:** Bruno Romeira, Luis M. Pessoa, Henrique M. Salgado, Charles N. Ironside, José M. L. Figueiredo

**Affiliations:** 1 Centro de Electrónica, Optoelectrónica e Telecomunicaçõe s (CEOT), Departamento de Física, Universidade do Algarve, Campus de Gambelas, 8005-139 Faro, Portugal; E-Mail: jlongras@ualg.pt; 2 INESC TEC, Faculdade de Engenharia, Universidade do Porto, 4200-465 Porto, Portugal; E-Mails: luis.m.pessoa@inescporto.pt (L.P.); hsalgado@inescporto.pt (H.S.); 3 School of Engineering, University of Glasgow, Glasgow G12 8LT, UK; E-Mail: Charles.ironside@glasgow.ac.uk

**Keywords:** double barrier quantum well structures, clock recovery, microwave-photonics, optical injection locking, optoelectronic circuits, oscillators, photo-detectors, radio-over-fiber, resonant tunneling diodes

## Abstract

We report on photo-detectors consisting of an optical waveguide that incorporates a resonant tunneling diode (RTD). Operating at wavelengths around 1.55 *μ*m in the optical communications C band we achieve maximum sensitivities of around 0.29 A/W which is dependent on the bias voltage. This is due to the nature of RTD nonlinear current-voltage characteristic that has a negative differential resistance (NDR) region. The resonant tunneling diode photo-detector (RTD-PD) can be operated in either non-oscillating or oscillating regimes depending on the bias voltage quiescent point. The oscillating regime is apparent when the RTD-PD is biased in the NDR region giving rise to electrical gain and microwave self-sustained oscillations Taking advantage of the RTD's NDR distinctive characteristics, we demonstrate efficient detection of gigahertz (GHz) modulated optical carriers and optical control of a RTD GHz oscillator. RTD-PD based devices can have applications in generation and optical control of GHz low-phase noise oscillators, clock recovery systems, and fiber optic enabled radio frequency communication systems.

## 1. Introduction

Semoconductor photo-detectors are devices that can convert optical signals into electronic signals, and they are extensively used in optical communication systems [1]. Ideally a photo-detector should detect all the incident light, respond to the fastest changes in the incoming signal and not introduce additional noise. In practice, photo-detectors have limited bandwidth with finite response times, they have associated noise sources and the probability of detecting an individual photon of light is less than one. Furthermore, photo-detectors have tradeoffs particularly between speed (bandwidth) and quantum efficiency, whereby designs are aimed to increase their bandwidth-efficiency product [1].

Several types of detectors can be used in fiber optic systems for communications, sensing and measurement systems. Some of the most common types include *p-i-n* photodiodes, metal semiconductor metal photodiodes and avalanche photodiodes [1]. Alternatives to traditional photo-detectors include optical control of microwave devices for generation of high-speed low-phase noise oscillations. Injection locking of optically controlled microwave oscillators allows synchronizing the frequency and phase of free-running oscillators to optical modulated signals and can find extensive applications, ranging from wireless communications [2] to high-speed optical communications such as radio-over-fiber networks [3,4], from bio-imaging for identifying cancers [5] to test instruments. Direct optical control of microwave devices has been investigated in Gunn oscillators [6], tuning and power modulation of trapped plasma avalanche and transit time (TRAPATT) oscillators [7], and tuning of impact avalanche and transit time (IMPATT) oscillators [8]. However, these devices are limited in bandwidth and not amenable to integrated circuit realization and, because of nature of the processes involved, they require inconveniently high bias voltages, for example the avalanche effect is noisy and can introduce additional timing jitter and phase noise resulting in considerable inferior stability.

Currently, many researchers are looking for solutions to cleanly transfer the stability of high quality (*Q*) optical signals to microwave electronic signals. The proposed configurations employ either direct or external modulation of laser diodes ([9], and references therein), or heterojunction bipolar transistors (HBTs) [10], among others [11]. However, most of these schemes require several electronic and optoelectronic components such as phase-locked local oscillators, amplifiers, photo-detectors, light emitters, and/or amplifiers or modulators, which make them rather complex and costly for applications where compact solutions for the transmission and distribution of radio-frequency (RF) signals are needed [11].

Recently, optical injection locking of double barrier quantum well (DBQW) resonant tunneling diode (RTD) photo-detector devices to intensity modulated optical signals [12,13], and self-injection locking of a low cost, simple and compact RTD optoelectronic oscillator (OEO) configuration [14,15] have been successfully demonstrated that could substantially increase the practicality of optically controlled microwave oscillators for a wider range of microwave and photonics applications. In this article, DBQW-RTD based optical waveguide photo-detectors are described for the implementation of novel optically injection locked microwave oscillators that are controlled using signals at telecommunications wavelengths for applications in clock recovery and fiber optic based communication systems. The photodetection results show the noise of the microwave injection locked RTD signal matches that of the input optical analogue and digital signals resulting in low timing jitter of the electrically detected carrier signal. Optically controlled RTD-based photo-detectors can have interesting applications in generation of microwave stable low-phase noise signals for clock recovery systems, low-power miniature atomic clocks as time references [16], and synchronization in communication networks [17]. Because of the fast response of RTDs [18], this technology has the potential to deliver Gb/s wireless transmission [19] which matches the data capability of current fiber optics, and considerably enhances radio-over-fiber communications [20].

## 2. DBQW RTD Photo-Detectors

Double barrier quantum well RTD based photo-detectors are interesting alternatives for optical detection when compared with traditional photo-detectors due to their photo detection mechanism and built in electrical gain arising from RTD's current-voltage (I-V) characteristic showing a region of negative differential resistance (NDR). With optimum epi-layer structure design that takes advantage of a photo detection mechanism based on the scenario that in biased RTDs the photo-excited electron-hole pairs become locally separated, which in turn modulates the internal electric field, RTDs photo-detectors can have sensitivities of up to 10^3^ A/W [21]. By taking advantage of these effects, RTD structures can be used to implement light-by-light switching and optically switched RTD photo-detectors. Early demonstrations of such optically switched devices have shown potential for high-speed transmission communications at Gb/s rates with very low electrical power dissipation [22]. Demonstration of single photon detectors were also achieved combining DBQW-RTDs and quantum dot structures [23].

Resonant tunneling diode based photo-detectors (RTD-PDs) consist of nano-electronic structures that use vertical stacking of epitaxial layers of semiconductor alloys with the active regions consisting of 10-nm thick InGaAs/AlAs DBQW semiconductor alloys, surrounded by InGaAlAs photoconductive layers that act as like a Fabry-Pérot interferometer for the electron wave functions. The Fabry-Pérot effect gives rise to a highly nonlinear and N-shaped current *versus* voltage characteristic which provides the occurrence of NDR. DBQW-RTD structures for photo-detection emerged in the last decades as devices with high responsivities and gain-bandwidth-efficiency products, as well as low switching energies [23] because of their intrinsic gain and low voltage operation characteristics arising from the NDR effect. Essentially, they work as follows: a light pulse incident upon a resonant tunneling structure biased close to the peak voltage produces photo-charges that reduce the series resistance, leading to a shift of the peak and valley voltages which can induce RTD switching from the peak to the valley of I-V NDR and give rise to changes in the current flow [24].

### 2.1. Device Description

**Figure 1.**
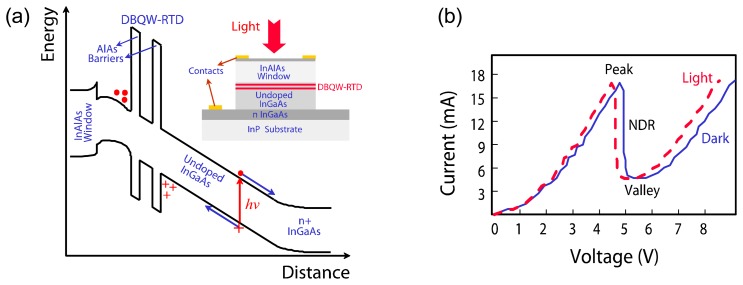
**(a)** Schematic of the conduction band diagram of a surface illuminated InGaAs/AlAs DBQW-RTD. Inset is shown the schematic cross-section of the surface RTD-PD [22]. (b) Typical dark and illuminated I-V characteristics.

An example of a surface-illuminated DBQW-RTD photo-detector consisting of InGaAs/AlAs semiconductor compounds is shown in Figure 1a. As exemplified in the conduction band diagram, since the applied external voltage drops almost entirely on the thick undoped InGaAs absorption layer, the photo-generated electrons and holes in it are separated by the electric field, the holes are accumulated at the barriers of the resonant structure whereas the electrons are collected by the external circuit. As a result, the applied electric field in the undoped layer is partially screened and that within the resonant structure is enhanced. Consequently, the NDR of the resonant tunneling device shifts to lower voltages, as exemplified in Figure 1b. If the accumulated holes have a long recombination time the sensitivity of the device is high but its frequency response is low whereas for fast hole recombination times the sensitivity is lower but high-speed can be achieved.

Side-illuminated waveguide photo-detectors were proposed as a means to ease the trade-off between the speed and responsivity in order to achieve a large bandwidth-efficiency product [25,26]. This type of detector is generally edge-illuminated with light guided parallel to the plane of the intrinsic layer and where the light is absorbed. The photo-generated carriers and the light travel in perpendicular directions. In comparison with the surface transverse photodiode, the waveguide longitudinal photodiode responsivity is mainly determined by the waveguide length, and not by absorption layer thickness, therefore the waveguide detector can have a larger bandwidth-efficiency performance than the surface photo-detector.

**Figure 2.**
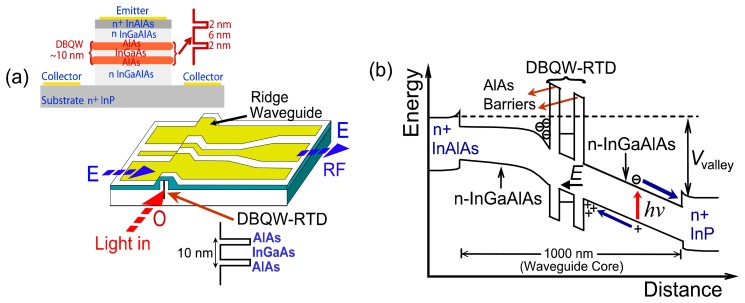
**(a)** Schematic diagram of RTD-PD device. The ridge waveguide dimensions were ∼3 μm wide and 1 μm thick. Inset (on top) is shown the schematic diagram (not scaled) of the RTD epi-layer structure, (b) RTD-PD unipolar In Al As-In_0.53_Ga_0.42_Al_0.05_ As-InP band energy diagram.

The optical waveguiding structure is similar to that of the double heterostructure lasers. Figure 2 shows the RTD-PD optical waveguide schematic, the wafer structure and the energy band diagram of the waveguide detector used in this work. The RTD component was a device fabricated from pre-existing material, the layer structure of which is given in [27]. The InGaAlAs RTD structure was grown by molecular bema epitaxy in a Varian Gen II system on a n^+^ InP substrate. It essentially consisted of two 2-nm-thick AlAs barriers separated by a 6-nm-wide InGaAs quantum well, embedded in a 1-μm thick InGaAlAs optical waveguide core, see epi-layer structure in the inset of Figure 2a. Besides the epi-layer structure shown in the inset of Figure 2a, a *δ*-doped InGaAs cap layer was provided for formation of Au-Ge-Ni ohmic contacts. Ridges were fabricated by wet-etching after which ohmic contacts were deposited on top of the ridges. A SiO_2_ layer was then deposited, and access contact windows were etched on the ridge electrodes making contact to high frequency bonding pads [coplanar waveguide (CPW) transmission line].

The design described here was originally used to obtain a DBQW-RTD operating as an electro-absorption modulator (EAM), but the same epitaxial structure can be used for novel photo-detector devices and applications as discussed in this article. Light is edge-coupled to the waveguide and the responsivity is mainly limited by the coupling efficiency and the scattering loss along the waveguide. The coupling can be improved through the use of lensed fibers, antireflection coating at waveguide facet, or laterally tapered waveguide sections.

### 2.2. Detection Mechanism in DBQW-RTD Waveguide Photo-Detectors

Inour device, the RTD operates as a waveguide photo-detector by taking advantage of DBQW-RTD photoconductive layers and optical waveguide structure. An RTD-PD optical waveguide works as a waveguide photo-detector for light with energy close or above the waveguide core bandgap energy. The ridge waveguide structure, Figure 2a, offers an advantage over traditional RTD-PDs by providing light confinement along the DBQW plane. The RTD-PD presents the typical RTD nonlinear I-V characteristic with an NDR region which leads to electrical gain. The photo-detection mechanism works as follows. For the low bias condition, electron-hole pairs are created in the depleted part of the waveguide core (that is, the 500 nm undoped In_0.53_Ga_0.42_Al_0.05_ As layer) adjacent to the double barriers, see energy band diagram of Figure 2b. However, due to the fact that the electric field is very weak under low bias, the generated electron-hole pairs tend to recombine and neutralize after the creations. As the bias is increased, the hole accumulation density at the hetero-interface increases, resulting in an enhancement of the hole tunneling current. However, for bias voltages below the peak voltage, the current flow is still low and therefore a portion of the photogenerated carriers recombine rather than tunnel through the DBQW structure. When the bias is further increased to the valley voltage, Figure 2b, the effective conductivity of the double barriers drops as the resonant level in the quantum well becomes lower than the Fermi level of the emitter region. Consequently, the band bending on the double barriers increase, resulting in a substantially increase of the hole tunneling current. Once the hole current becomes large enough, the recombination in the photogeneration region decreases to zero, and the photogenerated carriers are collected by the electrodes.

As described previously, if the photon energy is larger than the waveguide core band-gap energy (here 0.815 eV) the RTD-PD operates as a photoconductive detector. However, if the injected photon energy is slightly lower (e.g., 0.8 eV) the Franz-Keldysh effect [28], which is associated with interband photon-assisted tunneling, plays also a role in absorption mechanism, *i.e.*, the absorption of a photon is considerably enhanced due the presence of the electric field via the Franz-Keldysh effect.

### 2.3. DC Photo-Detection Characteristics

Photo-detection characterization of RTD-PDs consisted in coupling light into the waveguide cleave ends (endfire coupling) using a lensed single mode optical fiber (spot diameter of 5 ± 0.5 μm and coated on the tip). The lensed fibers and the RTD-PD devices were mounted on microblock 3-axis translation stages, in an endfire arrangement where both input optical fibers and samples could be translated and rotated independently. The light source employed for optical detection characterization was a Photonetics Tunics laser diode tunable in the wavelength region 1,457–1,599 nm, with up to 10 mW optical output power, that could be used for high frequency modulation with a bandwidth up to 1 GHz. RTD-PD devices mounted on the brass fixture were characterized directly on-chip by probing the RTD CPW contacts using a ground-signal-ground CPW probe with a pitch size of 125 μm.

**Figure 3.**
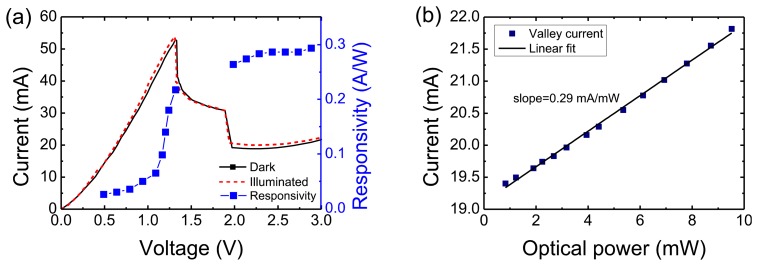
**(a)** Dark RTD-PD I-V characteristic and I-V curve with illumination at 1,550 nm for input optical power in fiber around 5 mW. The estimated responsivity as a function of the bias voltage is also plotted. (**b**) Photogenerated valley current as a function of illumination intensity and the linear regression.

Due to the polarization dependence of the RTD waveguide detectors, laser light was coupled to the waveguide using a polarization controller and the position of the input fiber was optimized to obtain the highest photocurrent from the device (and hence a high response to light modulated signals), ensuring a maximized overlap between the optical mode from the input fiber and the DBQW region. In Figure 3a is shown the I-V characteristics for both dark and illuminated conditions showing the photocurrent gradually turned on as the bias was increased to the valley voltage. Consequently, the NDR of the RTD-PD shifts to lower voltages, although the effect of the current increase shown in Figure 3a is not very pronounced because of the low optical power received by the RTD due to the small coupling efficiency as detailed next in this sub-section. Also shown is the external responsivity (the photocurrent generated divided by the optical power in fiber) as a function of the bias voltage. Note that a bias voltage of only 0.5 V was required to efficiently collect the photogenerated carriers. The results show clearly a higher responsivity close in or in the valley region which confirms the detection mechanism analyzed previously. Figure 3b presents the photogenerated valley current plotted *versus* the optical power in fiber. The straight line in the figure is the linear regression obtained from the data. The photogenerated valley current increases approximately linearly with the illumination power level (*dI/dP_i_*) with a slope of 0.29 mA/mW, and up to 10 mW in-fiber optical power level. No saturation of the photogenerated valley current was observed, indicating that photoexcited RTD waveguide detectors can operate at moderate incident power light conditions.

The quantum efficiency in photo-detectors is defined as the fraction of photons creating electron-hole pairs:

(1) $$\eta_{ph}=\frac{I_{ph}}{q\phi }$$

where *I_ph_* is the photocurrent, and *φ* = *λP*(*λ*)*/hc* is the photon flux, with *λ* the operation wavelength, and *h* and *c* being the Planck constant and the speed of light in the vacuum, respectively. Therefore, the RTD photo-generated current *I_ph_* in response to an incident optical signal *P*(*λ*) can be expressed by:

(2) $$I_{ph}=\eta_{ph}\frac{q\lambda }{hc}P\left(\lambda \right)$$

If the surface reflections and the finite dimensions of the RTD waveguide photo-detector are considered, then the quantum efficiency, *η_ph_* is given by light coupling factor *κ* multiplied by the absorbance [28]:

(3) $$\eta_{ph}=\kappa \left(1-R_{\text{ref}}\right)\left(1-e^{-\alpha \left(\lambda ,V\right)\gamma_{ph}\Lambda }\right)$$

with *R_ref_* is the waveguide facet reflectivity (sidewall imperfections and refractive index nonuniformity contribute to this kind of losses), *α*(*λ,V*) is the waveguide core absorption coefficient at wavelength *λ* and bias *V, γ_ph_* is the overlap integral of the electric field and the optical field, and Λ is the waveguide photo-detector absorbing active length. This expression does not take into account with propagation losses.

In Table 1 are presented the typical physical parameters of the RTD photo-detectors. For the waveguide structure described in Figure 2a, we estimate *γ_ph_* ∼0.25 and *κ* ∼0.35 at 1.55 μm, and an absorption coefficient *α*(*λ,V*), in the valley region, around 400 cm^−1^, estimated from absorption spectra characterization reported in [11], which for devices with absorbing active length A ≥ 150 μm yields a quantum efficiency *η_ph_* ∼0.2. Finally, the external responsivity is expressed by:

(4) $$R_{\lambda }=\frac{\lambda }{1.24}\eta_{ph}$$

that gives a responsivity of 0.25 AAV in the valley region, which compares well with the typical values measured between 0.25 AAV and 0.3 AAV for RTD-PD devices biased in the valley region (see measured responsivity in the valley region shown in Figure 3a). According to the calculations, most of the light is not coupled in the waveguide (25% coupling efficiency which gives a coupling loss of 6 dB), which seems to be the major factor limiting the responsivity.

Although the responsivity of 0.25 AAV at these wavelengths (∼1,550 nm) is low and below available commercial devices, as shown in the next section, the values reported here are sufficient to achieve an efficient optical control of the RTD microwave signals when the RTD is operating in the oscillating regime. Moreover, considering the estimated losses, we expect RTD-PD devices can provide PDs with responsivities up to 1 A/W which will further improve the efficiency of the optical control. The losses can be reduced by increasing the overlap integral of the input beam profile with the waveguide cross-section, reducing facet reflections, and design an optical waveguide with the RTD-PD active region closer to the input facet. In addition, calculations show that there would be no significant increase in the responsivity even with a much higher overlap, indicating that ∼150 μm is long enough to absorb almost all light. In fact, calculations suggest there is not a substantial increase in responsivity for waveguides longer than ∼100 μm. Therefore, the waveguide structure will occupy much less area, and the RTD could operate at higher frequencies. Currently, we are also investigating RTD-based surface illuminated photo-detectors which can provide advantages in terms of overlap integral of the input beam profile and coupling efficiency.

## 3. Regimes of Operation of DBQW RTD Photo-Detectors

The RTD-PD can be operated in either non-oscillating or oscillating regimes depending on the bias voltage quiescent point. The oscillating regime of operation takes place when the RTD-PD is biased in the NDR region giving rise to electrical gain and microwave self-sustained oscillations which boosts RTD sensitivity to incident modulated light signals. In this section, we demonstrate efficient detection of GHz modulated optical carriers and optical control of RTD high-frequency oscillator when RTD-PD devices are operated in the non-oscillating and oscillating regimes.

### 3.1. Non-Oscillating Regime

The measurement system used for the characterization of the RTD-PD optical modulation response in the non-oscilating regime consisted of continuous wave light from a tunable single mode laser modulated by an external 10 GHz electro-optic modulator driven by an RF signal generator. A single mode optical fiber was used to guide the modulated light and to illuminate the RTD-PD waveguide by means of a lensed fiber. The RTD-PDs, the electrical GSG probe (with high frequency response up to 40 GHz), and the optical fiber were mounted on the positioners as described previously in the DC characterization setup. The variable DC bias was applied via a wide bandwith bias-T (45 MHz–26.5 GHz) connected to the high-frequency probe SMA cable. The RTD-PD electrical response to optical modulated injected signals was measured using a high-bandwidth spectrum analyzer and oscilloscope.

**Figure 4.**
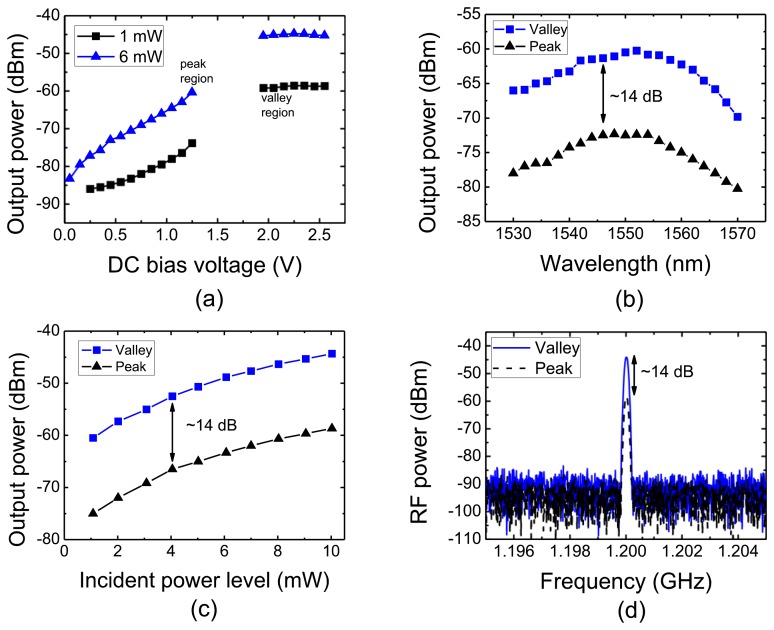
**(a)** RF injection locking capture level as function of the DC bias using light at *λ* = 1,550 nm. (**b**) Output power as function of wavelength with DC bias voltage as parameter for 1 mW optical power in fiber. (c) Output power as function of optical power level at *λ* = 1,550 nm. (**d**) Power spectra of photo-detected signal using a 10 mW optical signal *X* = 1,550 nm). In figures (**b**), (**c**), and (**d**), the RTD-PD was DC biased in the peak *V_p_* = 1.2 V and valley *V_v_* = 2.0 V regions. In all figures, light was modulated by a RF reference source signal at 1.2 GHz.

Figure 4a presents the RF injection locking capture level as function of the DC bias using light (*λ* = 1,550 nm) modulated by a sinusoidal RF reference signal at 1.2 GHz, with optical power levels in fiber of 1 mW and 6 mW.

The figure shows the dependence of 1.2 GHz photodetection signal on bias conditions. The responsitivity increases with the transition from RTD-PD peak to valley voltage, *V_p_* and *V_v_*, respectively, with an improvement up to 14 dB. As discussed previously, the higher response in this region occurs because the holes photo-generated in the depletion region accumulate around the collector barrier (see Figure 2b), causing a local enhancement field which leads to higher carrier velocity and hence high current. Figure 4b presents the photo-detected RF power as function of the light wavelength with the DC bias voltage as parameter. Again, the increased responsivity under higher voltage bias has also been observed at the wavelength of 1,530-1,570 nm (C-band).

Figure 4c presents the measured RTD-PD RF power output when biased in the peak and valley regions as a function of incident optical power, and Figure 4d shows the RF power spectra of the photo-detected optical modulated signals. Up to 14 dB gain is obtained in the transition from the peak to the valley region. The results demonstrate the RTD-PD works as a high-speed optical-to-electrical converter, taking advantage of the optical waveguide design. In what follows, the optical injection locking operation employing RTD-PDs is investigated when the devices are DC biased in the NDR region, *i.e.*, when they generate self-sustained oscillations at frequencies close to the detected microwave signals.

### 3.2. Oscillating Regime and Optical Control of Microwave Signals

Optical injection locking of an electrical oscillator can synchronize the frequency and phase of a free-running oscillator to the modulated optical signal. This, is very attractive in applications such as local oscillators embedded in radio base station cells and in optical transmission systems, e.g., optoelectronic clock recovery circuits, because it can lead to much simpler systems and is more suitable for higher bit-rates than a fully electrical circuit. There are two types of optically injection locked oscillators (OILOs): the direct and indirect type. A direct OILO, whose active oscillator device itself is directly illuminated for synchronization is preferred because is much simpler and is more suitable for monolithic integration than an indirect one which needs an external photo-detector. In this section, we investigate the injection locking phenomena of an RTD-PD circuit showing self-sustained oscillations up to 1.41 GHz. The influence of optical injection locking on the quality of the microwave carriers is evaluated and we achieved phase noise quality of the signals that are of interest for many microwave photonics applications, as discussed in detail in Section 4.

**Figure 5.**
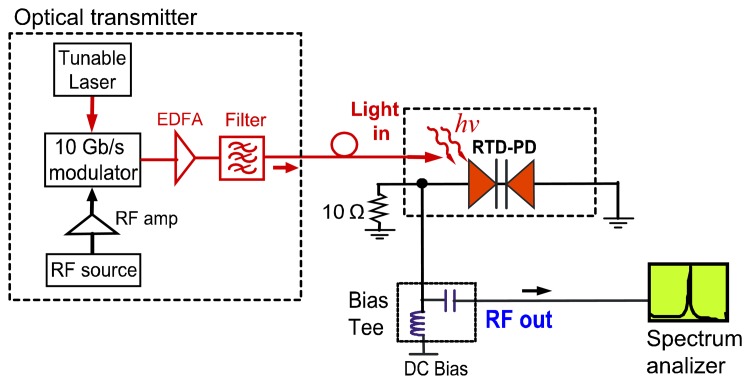
RTD-PD oscillator circuit schematic, and setup of the optical injection locking experiment.

The RTD-PD based oscillator circuit utilized in the optical injection locking experiments was realized using RTD components attached directly onto the surface of a microwave substrate containing a 50 Ω copper microstrip transmission line laminated onto a non-conductive substrate. The transmission lines were realized in microstrip hybrid technology using Rogers substrate with a dielectric constant of 3.48 and a thickness of 0.762 mm. At the end terminals of the 50 Ω microstrip line were high frequency SMA connectors which acted as the RF output port. Gold bond wires were employed to connect the RTD-PD emitter and collector to the 50 Ω transmission line and to the ground, respectively. The RTD-PD devices were attached to the substrate with a high conductivity adhesive. The orientation of the RTD's optical waveguide was taken into account in the layout and in the chip bonding to PCB to ensure good alignment to an optical fiber. In the oscillator circuit, a 10 Ω thin-film resistor, Figure 5, was utilized as part of the bias circuit of the oscillator to suppress the bias circuit spurious oscillations for the RF signals generated by the RTD [14]. The operation frequency of the RTD-PD is essentially determined by the RTD-PD's parallel capacitance and the series equivalent inductance of the wire bonding connections (the wire length was around 4 mm, giving an equivalent inductance around 3.6 nH), which for the circuit reported here gives an operation frequency up to 1.4 GHz. High-frequency characterization of these devices was previously investigated [29] providing detailed analysis of RTD impedance *versus* frequency dependence for various bias voltage values. For a 45 MHz to 18 GHz frequency range the samples with negative resistance *R* ∼ −15Ω. (estimated from the NDR region) showed RTD equivalent capacitance of RTD around 1.5 pF. For these circuit parameters, one can estimate the typical maximum frequency of operation of RTD devices, *f_max_* ∼14.15 GHz. Therefore, the RTD-PD hybrid circuit oscillators employed in our experiments could operate up to 10 GHz reducing circuit parasitics.

The circuit diagram and experimental setup are presented schematically in Figure 5 showing the optical transmitter and measurement setups. Optical modulated signals at communications wavelengths were generated by a 10 Gb/s Mach-Zehnder modulator that encode the light (typically λ = 1,550 nm) from a tunable single mode laser. The output from the Mach-Zehnder modulator was amplified by an erbium-doped fiber amplifier (EDFA), and then filtered to eliminate spontaneous emission noise from the EDFA. Then, optical modulated signals illuminated the RTD-PD oscillator waveguide input facet through single mode lensed fiber. The free running oscillation frequency of the RTD-PD oscillator was controlled by adjusting the bias voltage provided by a power supply and through an external bias tee. Optically injection locked signals were measured in the electrical output of the circuit using a high frequency oscilloscope and spectrum analyzer.

In the following, we study the injection locking phenomena of an RTD-PD circuit showing an oscillation ranging from 1.04 GHz to 1.41 GHz as the DC bias is swept across the NDC region with voltage range ∼0.4 V. The coupling of optical modulated signals to the RTD-PD waveguide locks the self-oscillations to the optically injected RF sub-carrier for in-fiber optical powers above a given threshold that depends on the waveguide characteristics, on the frequency and the RF power of the modulated light, and on the RF power of the unperturbed self-oscillations. To investigate the influence of optical injection locking on the quality of the RF carriers, signals were observed at the output RF port of the RTD-PD oscillator. The oscillator was considered injection-locked when its output spectrum matched the optical modulation reference source frequency with stable oscillation and minimum noise.

**Figure 6.**
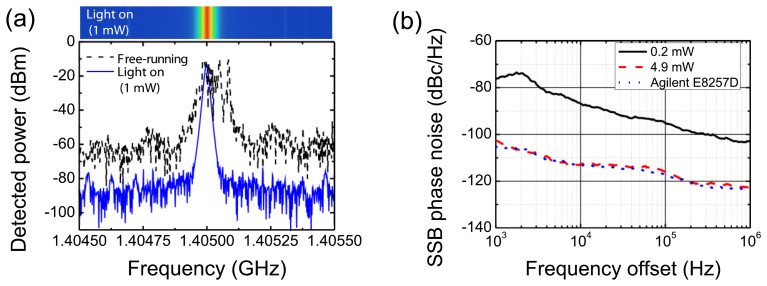
**(a)** Power spectra of the free-running and locked signals. (**b**) Single side band phase noise measurements.

The optical injection locking investigation included measurements of a RTD-PD circuit self-oscillating at around 1.405 GHz. The circuit showed a substantial improved performance in terms of injection locking and spectral purity. The results are summarized in Figure 6. Figure 6a presents the typical broad spectrum of free-running oscillation (light off) around 1.405 GHz. When the light is on, with an average optical power level in fiber of 1 mW and employing a modulation extinction ratio close to 10 dB, the free-running signal is pulled and the spectrum collapses showing a substantial phase noise reduction. The inset of Figure 6a (top) presents the recorded spectrum surveillance confirming the spectral long-term stability of the optical injection locking. Injection locking for optical power levels in fiber as low as 0.2 mW was observed. The locking range was 23.8 MHz for 4.9 mW optical power in fiber. This corresponds to an exceptional wide value with a fractional locking bandwidth of 1.7%. The observed locking range corresponds to the largest value reported in the literature for RTD-based devices at communication wavelengths ([12] and references therein).

Since injection-locking leads to a significant phase-noise and jitter reduction the respective oscillator performance has been further investigated. Figure 6b presents the phase noise measurements showing phase noise of −112.4 dBc/Hz at 10 kHz offset of the carrier frequency, a very close match to the RF source (Agilent E8257D) signal for the moderate optical power levels employed (below 5 mW), which results in a noise suppression of more than 40 dB when compared with the free-running oscillator without illumination. For the infiber optical power locking threshold the phase noise was −87.1 dBc/Hz at 10 kHz offset of the carrier frequency.

The results, although obtained using devices with small responsivities when compared with commercial available photo-detectors, show the RTD-PD can operate as a stable optically controlled microwave photonics oscillator to obtain a highly efficient O/E conversion. The RTD-PD phase noise characteristics should be sufficient for many current microwave photonic applications that use phase modulated schemes, e.g., phase shift keying (PSK) data transmission (as discussed in the next section), as long as the locking conditions are achieved and maintained.

## 4. Applications in Clock Recovery and Fiber Optic Supported Radio Frequency Communications

In this section, we demonstrate applications of injection locked RTD-PD oscillator devices in microwave photonics systems. The ability to lock to reference RF sources by optical injection locking techniques allows remote synchronization providing a large range of functions including generation, amplification and distribution of RF carriers, clock recovery, carrier recovery, modulation, and frequency synthesis. Optically controlled RTD-based photo-detectors can have interesting applications in the generation of microwave stable low-phase noise signals ranging from wireless communications [17] to high-speed optical communications such as radio-over-fiber networks [20], from clock recovery systems to low-power miniature atomic clocks as time references [16]. In what follows two main applications of RTD-PD oscillators in high-speed optical communication transmission systems are discussed: clock-recovery of Gb/s return-to-zero signals and transmission of modulated signals in wireless/photonics links.

### 4.1. Clock Recovery

The ability to recover the clock signal from incoming random data is a fundamental operation in digital communications. Clock recovery consists in deriving a clock signal from the received signal which is synchronized in both frequency and phase, avoiding its transmission along with the data, and providing a timing basis to sample the received signal. In this context, the ability to perform clock recovery using optical techniques is an important issue in the development of circuits for novel high speed optical communication systems.

RTD-PD oscillators are candidates for that purpose since they combine the functions of microwave oscillation and photo-detection, while offering intrinsic gain and high responsivities, being suitable for injection locking with incoming data in both optical and electrical domains. In what follows we present results on a clock recovery experiment using optical injection (O/E conversion). Clock recovery via optical injection is important because it enables the clock of a high-speed data signal in a fiber-optic system to be directly recovered without first converting the data to electrical pulses. In this experiment the free running RTD-PD oscillator is tuned to oscillate at a frequency sufficiently close to the incoming signal. The locking range is typically on the order of tens of MHz, and depends on the power of the injected clock frequency. The incoming optical signal is injected into the oscillator and the recovered signal is accessed at the electrical output port.

**Figure 7:**
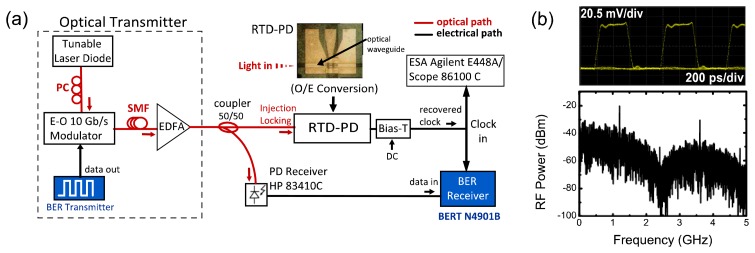
**(a)** Experimental schematic for timing extraction and BER measurements via optical injection locking. (**b**) RZ data stream input of 1.24 Gb/s with 100 mV amplitude (V_p-p_) and 50% duty cycle using a PRBS of 2^7^ − 1; (top): eye diagram of RZ data; (bottom): RZ data signal measured in the frequency domain.

Figure 7 shows the experimental schematic for timing extraction and bit-error-rate (BER) measurements. The performance of the recovered clock was assessed from timing jitter, phase noise and locking bandwidth. Additionally, BER measurements were carried out using the recovered clock as a time base for sampling of the received data.

Clock recovery and error free timing extraction results are demonstrated using return-to-zero (RZ) format data signals that optically injection locks the free-running RTD-PD oscillator. A 1.24 Gb/s RZ signal generated from a bit-error-rate test (BERT) transmitter, whose spectrum together with the respective eye diagram are shown in Figure 7b, was used to modulate a tunable laser diode optical carrier at 1.550 nm using a 10 Gb/s modulator that then fed an EDFA which output was subsequently split in a 50/50 coupler. One of the coupler outputs went to a commercial photo-receiver whose output was connected to the error analyzer. The other part of the optical signal was coupled to the RTD-PD optical input, Figure 7a.

**Figure 8.**
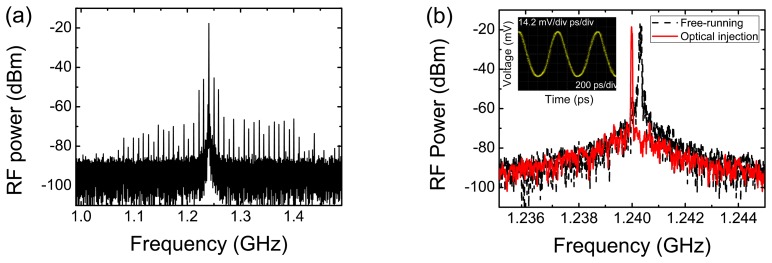
**(a)** The 1.24 GHz clock recovery in the frequency domain from an optically injected RZ data signal using an in fiber optical power level of ∼3 mW. (**b**) Power spectra of the free-running signal and the 1.24 GHz recovered clock (span of 10 MHz). The inset shows the recovered clock in the time domain.

Figure 8a shows the frequency spectrum of the data after clock recovery via optical injection of a 3 mW in fiber optical power signal, showing a large frequency component at the clock frequency. Figure 8b presents the power spectra of the free-running oscillation slightly above 1.24 GHz and the injection locked output at 1.24 GHz. The output spectrum was clearly pulled to 1.24 GHz in the presence of the optical injected signal.

**Figure 9.**
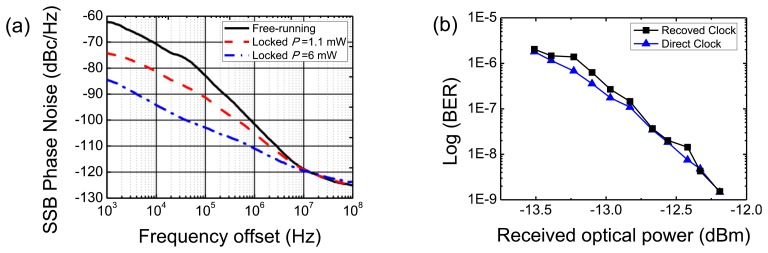
**(a)** SSB phase noise of free-running and recovered clock signals. (**b**) BER measurements of recovered and direct clocks.

The inset of Figure 8b presents also the injection locked RTD-PD output signal in time domain. A phase noise reduction of around 24 dB at 10 kHz offset from the carrier frequency was achieved for an optical injection power level in fiber of around 6 mW, Figure 8a. The minimum optical power in the fiber to obtain locking was around 1.1 mW with a locking range of 0.3 MHz. The locking range and the phase noise reduction were strongly dependent on the power of the optically injected signal. The phase noise was considerably reduced as shown in Figure 9a. The RMS jitter measured by the oscilloscope was 2.16 ps, which also matches well with that from phase noise measurement. The integrated timing jitter over a 90-kHz bandwidth was below 2 ps.

A comprehensive way to characterize the performance of a recovered clock signal is to measure the BER. First, the quality of data detection was investigated for a direct clock situation where the clock signal from the pattern generator was used to trigger the BERT equipment. Next, without illumination, because of the drifting of the RTD oscillation frequency it was not possible to provide a clock signal to trigger the BERT instrument. Then, after illumination the optical injection locking mechanism provided a stable oscillation reference source and clock recovery was performed where the optically injection locked RTD-PD electrical output was used to trigger theBER equipment. Figure 9b shows the BER at 1.24 Gb/s as a function of the received power for both direct and recovered clocks using a data pattern length of 2^7^ − 1. The two BER curves are similar, confirming the low jitter of the extracted clock signal. It is believed that the mismatch in some of the BER points results from environmental instabilities (vibration, temperature fluctuations) which can be reduced significantly with further optimizations in the measurement setup.

In summary, clock recovery using RTD-PD oscillators was demonstrated by taking advantage of the optical injection locking capabilities of the devices. One of the attractive properties include the power of the recovered signal is independent of the input power of the signal to be recovered. Other attractive properties include frequency tunability and relatively wide tracking range.

### 4.2. Fiber Optic Supported Radio Frequency Communications

The demand in the coverage and speed of optical communications networks, together with rapid growth in wireless networks, has motivated an increasing attention into hybrid wireless-photonics communication systems. The integration and transmission of RF signals over fiber, RoF systems, are expected to play an important role in broadband wireless communication systems. Taking advantage of optical fiber low loss and wide bandwidth transmission characteristics, it is possible to distribute broadband data and/or high frequency signals to many wireless subscribers. Among several approaches for realizing fiber optic supported radio frequency communication systems, many efforts has been dedicated to providing low cost and simple radio access points bringing the fixed network bandwidths closer to the mobile users [3,4].

Digital standards based on phase modulation, such as Gaussian minimum shift keying (GMSK), quadrature phase shift keying (QPSK), and quadrature amplitude modulation (QAM) are the basis for communications through the latest generation of mobile telephones and wireless local area networks (LAN). In this subsection, results on the transmission of digital modulated signals are demonstrated using a wireless/photonics arrangement that take advantage of the optical injection locking characteristics of RTD-PD oscillator circuits, and the error vector magnitude (EVM) performance of the RTD-PD for different phase and frequency modulation formats are evaluated, namely phase shift keying (PSK), Gaussian minimum shift keying (GMSK) and frequency shift keying (FSK). The investigation presented here allows us to assess the implementation of RTD-PD-based oscillator transceivers as simple and compact low phase-noise oscillators for optical/electrical signal processing units in fiber optic supported radio frequency communications and cellular network base stations. Although in this work we operate at 1-2 GHz frequencies partly because the signals are easier to analyze and the data rates easier to measure with the equipment available in our laboratories, the principles we present could be replicated at higher frequencies [18].

**Figure 10.**
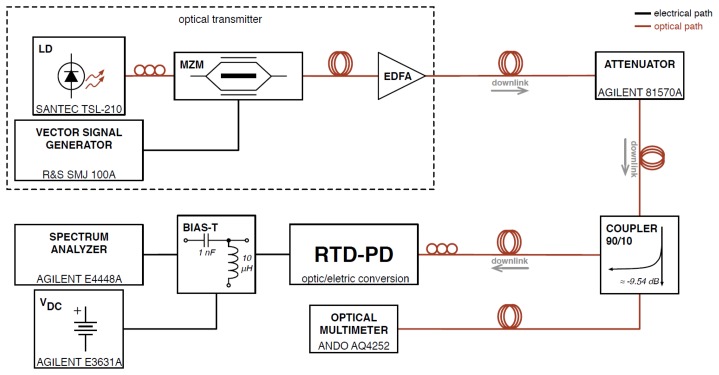
Description of the downlink experimental setup.

The setup of an optical-RoF downlink is shown schematically in Figure 10. The vector signal generator (VSG) signal was used to modulate an optical carrier generated by a tunable laser diode at 1,550 nm using a 10 Gb/s Mach-Zehnder modulator. The modulated optical signal was subsequently amplified by an EDFA, then fed to a variable optical attenuator, and then split in a 90/10 coupler. One of the coupler outputs went to an optical multimeter used for controlling the power level. The other part of the optical signal was coupled to the RTD-PD ridge waveguide, which locks to the carrier modulated on the incoming optical signal and converts it to the electrical domain, with responsivity around 0.25 AAV. Finally, a power supply provides the necessary DC biasing and a spectrum analyzer downconverts the received signal to be demodulated by the Agilent 89600 VSA software.

**Figure 11.**
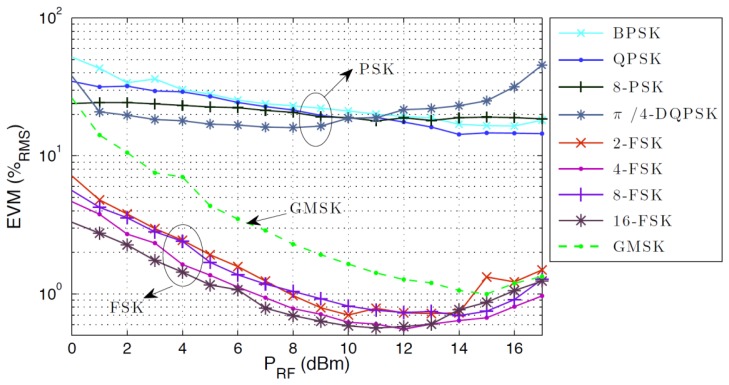
EVM performance comparison for PSK, FSK and GMSK modulated formats for the downlink.

The locking range was measured as a function of the injected optical carrier power. It was found to be in average {2, 4.2, 6.5, 9} MHz, for injected optical powers of {−0.5, 3.5, 6.5, 9.5} dBm, respectively. The power of the electrical carrier on the VSG was kept at 17 dBm, and we verified that the locking range also depends significantly on this parameter. The results show that the higher the power of the modulating electrical carrier or the injected optical carrier the larger the locking range. The measured electrical output power of the RTD-PD was −7.9 dBm. The measured performance for different modulation formats is shown in Figure 11, where the EVM was obtained as a function of the power of the electrical carrier modulation, using a data-rate of 2 Msymbols/s, for a fixed injected optical power of 9.5 dBm. The PSK modulation formats provide EVM results no better than 14%, while FSK based formats achieve 0.55% and GMSK attains 1% in the best case, which is again one order of magnitude better. It can be observed that the downlink performance is generally affected by the limitations associated to the optical injection process itself, since the coupling method is still very incipient, it could be optimized in a future prototype, for example by including facets with anti-reflection coatings, or tapering the ridge end region. The poor efficiency of the optical coupling leads us to the necessity of amplifying the optical signal, which introduced noise and consequently degraded the EVM results. Furthermore, there is observable performance degradation for electrical modulated powers above 10 dBm, which results from the nonlinearity in the Mach-Zehnder modulator.

As a summary, we have determined the error vector magnitude performance of different phase and frequency modulation using RTD-PD based oscillator circuits. We conclude that frequency modulated formats achieve better performance, although the Gaussian Minimum Shift Keying format also appears to be a practical alternative. Furthermore, future activities may address the performance of the device under realistic simultaneous downlink traffic, as well as the optimization of the optical injection efficiency.

## 5. Conclusions

We have presented double barrier quantum well resonant tunneling diode based optical waveguide photo-detectors for the implementation of novel optical injection locked microwave oscillators. These novel devices operate at optical communication wavelengths. We have demonstrated efficient detection of GHz modulated optical carriers and optical control of RTD high-frequency oscillator when RTD-PD devices are operated in the non-oscillating and oscillating regimes. When operating in the oscillating regimes clock recovery from return-to-zero format data by using injection locking of an RTD-PD free-running oscillator circuit was successfully demonstrated. Finally, transmission of digital modulated signals was demonstrated using a wireless/photonics arrangement that takes advantage of the optical injection locking characteristics of RTD-PD oscillator circuits. The error vector magnitude performance of the RTD-PD for different phase and frequency modulation formats was evaluated.

RTD-PDs provide simple and compact circuit configurations and can find extensive applications in high-speed optical communications such as synchronization in communication networks and radio-over-fiber links. RTDs are very high-speed devices and we anticipate that the systems and devices we have presented here could be implemented at much higher frequencies.

## Figures and Tables

**Table 1.** Description of the typical physical parameters of the waveguide photo-detector.

**Symbol**	**Quantity**	**Typical order of magnitude**
*λ*	Operation wavelength	1.55 μm
*κ*	Light coupling factor	0.35
*R_ref_*	Waveguide facet reflectivity	0.3
*α*(*λ,V*)	Waveguide core absorption coefficient (at 1.55 μm and biased in the valley)	400 cm^−1^
*γ_ph_*	Overlap integral of the electric and optical fields	0.25
Λ	Waveguide contact length	150 μm

## References

[1] Sackinger E. (2005). Broadband Circuits for Optical Fiber Communication.

[2] Granastein V. L. (2012). Physical Principles of Wireless Communications.

[3] Sauer M., Kobyakov A., George J. (2007). Radio over fiber for picocellular network architectures. J. Lightware Technol..

[4] Gomes N.J., Morant M., Alphones A., Cabon B., Mitchell J.E., Lethien C., Csornyei M., Stohr A., Iezekiel S. (2009). Radio-over-fiber transport for the support of wireless broadband services. J. Opt. Netw..

[5] Klemm M., Leendertz J.A., Gibbons D., Craddock I.J., Preece A., Benjamin R. (2009). Microwave radar-based breast cancer detection: Imaging in inhomogeneous breast phantoms. IEEE Ant. Wirel. Propag. Lett..

[6] Guetin P. (1969). Interaction between a light beam and a Gunn oscillator near the fundamental edge of GaAs. J. Appl. Phys..

[7] Kiehl R.A. (1978). Behavior and dynamics of optically controlled TRAPATT oscillators. IEEE Trans. Electron Devices.

[8] Yen H.W., Barnoski M.K., Hunsperger R.G., Melville R.T. (1977). Switching of GaAs IMPATT diode oscillator by optical illumination. Appl. Phys. Lett..

[9] Lee K.H., Kim J.Y., Choi W.Y., Kamitsuna H., Ida M., Kurishima K. (2008). Low-cost optoelectronic self-injection-locked oscillators. IEEE Photonic Technol. Lett..

[10] Shumakher E., Magrisso T., Kraus S., Elias D.C., Gavrilov A., Cohen S., Eisenstein G., Ritter D. (2008). An InP HBT-based oscillator monolithically integrated with a photodiode. J. Lightwave Technol..

[11] Seeds A.J., Williams K.J. (2006). Microwave photonics. J. Lightware Technol..

[12] Ramond T., Hollberg L., Juodawlkis P.W., Calawa S.D. (2007). Low noise optical injection locking of a resonant tunneling diode to a stable optical frequency comb. Appl. Phys. Lett..

[13] Romeira B., Figueiredo J.M.L., Ironside C.N., Kelly A.E., Slight T.J. (2010). Optical control of a resonant tunneling diode microwave-photonic oscillator. IEEE Photonic Technol. Lett..

[14] Romeira B., Seunarine K., Ironside C.N., Kelly A.E., Figueiredo J.M.L. (2011). A self-synchronized optoelectronic oscillator based on an RTD photo-detector and a laser diode. IEEE Photonic Technol. Lett..

[15] Romeira B., Javaloyes J., Figueiredo J.M.L., Ironside C.N., Cantú H.I., Kelly A.E. (2013). Delayed feedback dynamics of liénard-type resonant tunneling-photo-detector optoelectronic oscillators. IEEE J. Quantum Electron..

[16] Knappe S., Shah V., Schwindt P.D.D., Hollberg L., Kitching J., Liew L.-A., Moreland J. (2004). Microfabricated atomic clock. Appl. Phys. Lett..

[17] Romeira B., Figueiredo J.M.L., Slight T.J., Wang L., Wasige E., Ironside C.N., Kelly A.E., Green R. (2009). Nonlinear dynamics of resonant tunneling optoelectronic circuits for wireless/optical Interfaces. IEEE J. Quantum Electron..

[18] Wang J., Wang L., Li C., Romeira B., Wasige E. (2013). 28 GHz MMIC resonant tunneling diode oscillator of around 1 mW output power. Electron. Lett..

[19] Ishigaki K., Shiraishi M., Suzuki S., Asada M., Nishiyama N., Arai S. (2012). Direct intensity modulation and wireless data transmission characteristics of terahertz-oscillating resonant tunnelling diodes. Electron. Lett..

[20] Cantú H.I., Romeira B., Kelly A.E., Ironside C.N., Figueiredo J.M.L. (2012). Resonant tunneling diode optoelectronic circuits applications in radio-over-fiber networks. IEEE Trans. Microware Theory.

[21] Hartmann F., Langer F., Bisping D., Musterer A., Hofling S., Kamp M., Forchel A., Worschech L. (2012). GaAs/AlGaAs resonant tunneling diodes with a GaInNAs absorption layer for telecommunication light sensing. Appl. Phys. Lett..

[22] Moise T.S., Kao Y.-C., Goldsmith C.L., Schow C.L., Campbell J.C. (1997). High speed resonant-tunneling photodetectors with low-switching energy. IEEE Photonic Technol. Lett..

[23] Li H.W., Kardynal B.E., Ellis D.J.P., Shields A.J., Farrer I., Ritchie D.A. (2008). Quantum dot resonant tunneling diode single photon detector with aluminum oxide aperture defined tunneling area. Appl. Phys. Lett..

[24] Moise T.S., Kao Y.-C., Garrett L.D., Campbell J.C. (1995). Optically switched resonant tunneling diodes. Appl. Phys. Lett..

[25] Chaisakul P., Marris-Morini D., Isella G., Chrastina D., Rouifed M.-S., Le Roux X., Edmond S., Cassan E., Coudevylle J.-R., Vivien L. (2011). 10-Gb/s Ge/SiGe multiple quantum-well waveguide photodetector. IEEE Photonic Technol. Lett..

[26] Fidaner O., Okyay A.K., Roth J.E., Schaevitz R.K., Yu-Hsuan Kuo, Saraswat K.C., Harris James S, Miller D.A.B. (2007). Ge–SiGe quantum-well waveguide photodetectors on silicon for the near-infrared. IEEE Photonic Technol. Lett..

[27] Figueiredo J.M.L., Ironside C.N., Stanley C.R. (2001). Electric field switching in a resonant tunneling diode electroabsorption modulator. IEEE J. Quantum Electron..

[28] Chuang S. L. (1995). Physics of Optoelectronic Devices.

[29] Alkeev N.V., Lyubchenko V.E., Ironside C.N., Figueiredo J.M.L., Stanley C.R. (2000). Super high-frequency characteristics of optical modulators on the basis of InGaAlAs resonance-tunnel heterostructures. J. Commun. Technol. Electron..

